# Review on nutritional composition of orange‐fleshed sweet potato and its role in management of vitamin A deficiency

**DOI:** 10.1002/fsn3.1063

**Published:** 2019-05-17

**Authors:** Satheesh Neela, Solomon W. Fanta

**Affiliations:** ^1^ Faculty of Chemical and Food Engineering, Bahir Dar Institute of Technology Bahir Dar University Bahir Dar Ethiopia

**Keywords:** antioxidants, orange‐fleshed sweet potato, polyphenols, sweet potato, β‐carotene

## Abstract

A wide variety of the roots and tubers plays a major role in human diet, animal feed, and industrial raw materials. Sweet potatoes (SPs) play an immense role in human diet and considered as second staple food in developed and underdeveloped countries. Moreover, SP production and management need low inputs compared to the other staple crops. The color of SP flesh varied from white, yellow, purple, and orange. Scientific studies reported the diversity in SP flesh color and connection with nutritional and sensory acceptability. Among all, orange‐fleshed sweet potato (OFSP) has been attracting food technologists and nutritionists due to its high content of carotenoids and pleasant sensory characteristics with color. Researchers reported the encouraging health effects of OFSP intervention into the staple food currently practicing in countries such as Uganda, Mozambique, Kenya, and Nigeria. Scientific reviews on the OFSP nutritional composition and role in vitamin A management (VAM) are hardly available in the published literature. So, this review is conducted to address the detailed nutritional composition (proximate, mineral, carotenoids, vitamins, phenolic acids, and antioxidant properties), role in vitamin A deficiency (VAD) management, and different food products that can be made from OFSP.

## 1 INTRODUCTION

Root and tuber crops play a significant role in agriculture and facilitate food security in many developing countries. In the year 2017 worldwide, 494.6 million tons of roots and tubers (including potato) are produced (FAOSTAT, 2019). Roots and tubers are part of diet for majority of the global population, with world average per capita consumption of 19.4 kg/year (2013–2015) and projecting to achieve 21.0 kg/year by 2025 (OCED‐FAO, 2016) and also contributing to animal feeds and industrial needs (starch source) (Scott, Rosegrant, & Ringler, 2000). Among the roots and tubers, SP (*Ipomoea batatas*) is very important after potato on the basis of production and consumption. SP is a dicotyledon associated with Convolvulaceae family and ranks worlds' seventh most important food crop (Ahn et al., 2010); it is a potential energy contributor and considered as fifth essential crop (fresh weight basis) after rice, wheat, maize, and sorghum (Ndolo, Nungo, Kapinga, & Agili, 2007). Currently, SP cultivation was reported in more than 115 nations (FAOSTAT, 2019), and SP was recognized as the secondary staple food and possess significant role in diet of humans in many underdeveloped countries (Van Jaarsveld et al., 2005). In contrast to the other staple food crops, Trancoso‐Reyes et al. (2016) defined that SP possess special attributes such as adoptability in wider topography, ability to grow in subsidiary circumstance, good productivity in short durations, and balanced nutritional composition. Sweet potato was reported to have good sensory acceptability due to the eye‐pleasing colors and sweet taste. This high sensory acceptability of some SP varieties was suitable in malnutrition management and facilitating food security in underdeveloped nations (Julianti, Rusmarilin, Ridwansyah, & Yusraini, 2017).

Central American countries are considered as the center of SP origin, but recent times they are extensively cultivated in the tropical, in the subtropical zone, and in few temperate locations with divergent agroclimatic conditions (Chandrasekara & Josheph Kumar, 2016). The roots of SP plants are primary storage organs and act as sinks to photosynthetic products, and it resulted in the deposition of different micro (phytochemicals)‐ and macro (carbohydrates)nutrients (Lemoine et al., 2013). SP was reported to have highest dry matter content due to its sink strength (Rukundo, Shimelis, Laing, & Gahakwa, 2013), and this crop was highly appreciated for the positive agronomic and nutritive characteristics (Abidin, Carey, Mallubhotla, & Sones, 2017).

**Figure 1:**
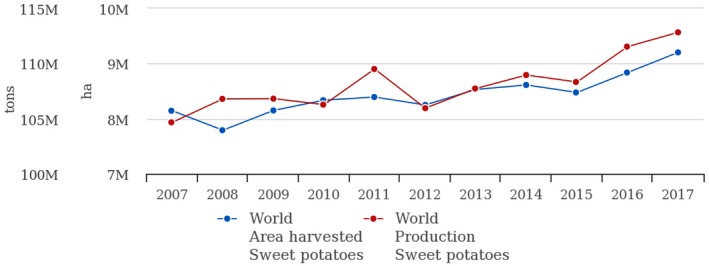
World area of harvested and production of sweet potato from 2017 to 2017 (source: FAOSTAT, 2019)

**Figure 2:**
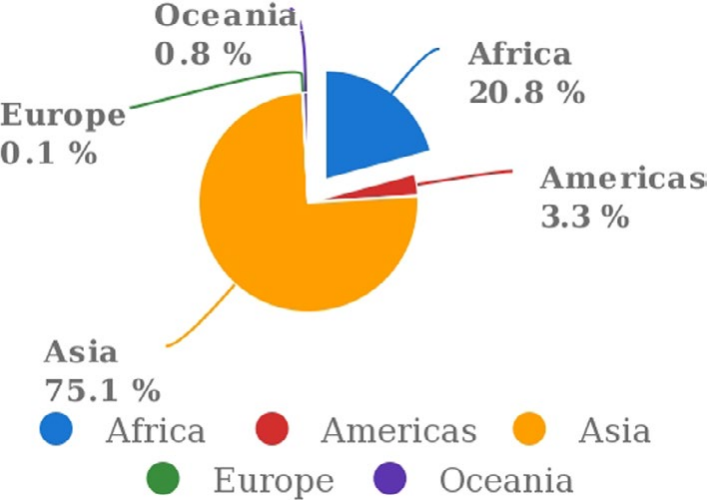
Production share of sweet potatoes by region from 2007 to 2017 (FAOSTAT, 2019)

Sweet potato production was reported to be 112.8 million tons (in 115 countries) in 2017, and China is the leading producer, followed by Nigeria and Tanzania, Indonesia, and Uganda (FAOSTAT, 2019). SP production and consumption in Africa, Asia, South American continents, and Caribbean islands are increased tremendously in recent times (Figures 1 and 2). SP is the most abundantly grown root crops in Africa. International Potato Center (2017) reported that sweet potato is 3rd vital food crop in seven central and eastern African countries, 4th priority crop in six South African nations, and 8th in four West African countries. SP is a key conventional crop, growing traditionally in limited area for domestic consumption purpose. SP is praised as a “poor man's” crop as it characteristically grown and consumed by meager communities especially by women‐headed families (Githunguri & Migwa, 2004; Ndolo et al., 2001). SP considered as the food security crop due to its low agriculture input requirements and high yields in wider climatic conditions (Ziska et al., 2009). SP crop is recently changing from a sustainable low‐input, low‐output crop to a significant cash crop. As a food security crop, it can be harvested at the point of demand as gradually (Tairo et al., 2005), also contributing to a reliable source of food and revenue to pastoral farmers who are frequently susceptible to regular crop damages.

Even though SP is a good source of carbohydrates (20%), the World Health Food Organization (WHFO) has acknowledged as the root crop with “antidiabetic” activity (Anbuselvi, Kumar, Selvakumar, Rao, & Anshumita, 2012). In vivo studies concluded that carbohydrate from SP stabilizes the sugar levels in blood and decreases the resistance to insulin (Mohanraj & Sivasankar, 2014). Hernández Suárez et al. (2016) reported that SP also provides the substantial quantities of selected vitamins (Vit C and PVA), specific minerals (potassium, magnesium, and calcium), and various bioactive compounds (phenolic acids and anthocyanins [ACN]) for consumers.

Researchers reported the clear role of variety difference in physical properties and chemical compositions of SP; for instance, van Jaarsveld, Marais, Harmse, Nestel, and Rodriguez‐Amaya (2006) reported that purple‐ and orange‐fleshed cultivars possess higher quantities of ACN and carotenes in comparison with white‐fleshed cultivars. In recent time, demand and attention on orange‐fleshed sweet potato (OFSP) are raised due to the high concentrations of β‐carotene (BC) and non‐pro‐vitamin A carotenoids (NPVAC). Kurabachew (2015) reported that OFSP has high potential to address VAD by food‐based intervention programs in targeted nations (Kurabachew, 2015). OFSP has given credit in recent scientific reports for its role in bakery, snack, and confectionery foods (Chen, Schols, & Voragen, 2003).

There are many scientific studies reported on the nutritional properties of the OFSP in different countries and varieties. Differences in nutritional contents are noticed among the varieties, and this created ambiguity in researchers. The review on the nutritional and bioactive composition of the OFSP is hardly available. So, this article is initiated to review the nutritional composition, bioactive components of OFSP, and role in VAD handling.

## 2 OFSP

From a dietary point of view and nutritional perspective, OFSP ranked as number 1 among all vegetables. OFSP tubers are considered as an significant dietary resource of VAC and NPVAC (Mohammad, Ziaul, & Sheikh, 2016). OFSP is appreciated due to the VA contribution and role in VAD eradication in developing countries (Girard et al., 2017; Kurabachew, 2015; Van Jaarsveld et al., 2005). Due to the many positive aspects related to agriculture, nutritional security and food security are resulted in intensified research on OFSP in present decade to augment its production and consumption in different countries. The OFSP possesses the characteristic of attractive sweet taste and eye‐pleasing yellow to orange color to children in comparison with white‐fleshed sweet potato (WFSP; Kaguongo, 2012); hence, OFSP has reported potential role to tackle calorific and VAD malnutrition problems of children in targeted communities.

Orange‐fleshed sweet potato is a good source of nondigestible dietary fiber, specific minerals, different vitamins, and antioxidants (Endrias, Negussie, & Gulelat, 2016; Rodrigues, Barbosa, & Barbosa, 2016). Phenolic compounds and carotenoids are responsible for distinguishing flesh and skin colors (deep yellow, red to orange, purple, and pale) of SP along with antioxidant properties (Steed & Truong, 2008). Scientists established the role of OFSP in health, and this accredited to its rich nutritional components with anticarcinogenic and cardiovascular disease (CVD)‐preventing attributes (Chandrasekara & Josheph Kumar, 2016; Jung, Lee, Kozukue, Levin, & Friedman, 2011). Recent scientific reports concluded the antioxidative and free radical scavenging activity of phenolic acid components in OFSP with beneficial health‐promoting activities (Bovell Benjamin, 2007; Rumbaoa, Cornago, & Geronimo, 2009; Teow et al., 2007). However, OFSP varieties with identical flesh color reported variations in phenolic content, individual phenolic acid profile, and antioxidant activity (AA) agents' concentrations. Reports on the OFSP incorporation in staple foods and its role in national food security and well‐being are readily available (Donado‐Pestana, Salgado, de Oliveira Rios, dos Santos, & Jablonski, 2012; Oki, Nagai, Yoshinaga, Nishiba, & Suda, 2006; Rumbaoa et al., 2009; Tang, Cai, & Xu, 2015; Teow et al., 2007).

## 3 NUTRITIONAL COMPOSITION OF OFSP

The OFSP is a good source of the basic nutrients and different vitamins, minerals, polyphenols, antioxidants, and ACN.

### 3.1 Proximate composition

Orange‐fleshed sweet potato is a second staple food crop in many countries of African and Asian continents for most of the population, so the proximate composition is very important to understand the role of this tuber crop in basic nutrition. In this section summarized is the proximate composition of the OFSP reported by different researchers. The proximate analyses of the OFSP from different countries and varieties are depicted in Table 1.

**Table 1:** Proximate composition of orange‐fleshed sweet potato reported by different authors

S. no.	Component	Rodrigues et al. (2016)^a^		$Huang et al. (1999)^b^	Endrias et al. (2016)^c^		Lyimo et al. (2010)^d^	Nascimento et al. (2015)^e^	Nicanuru et al. (2015)^f^	Mohammad et al. (2016)^g^
		$Fresh	Flour dried^h^	Fresh	Un pealed	Pealed	Dry weight	Dry weight	Wet base	Wet base
1	Moisture	69.42	10.97	62.2–78.2	76.97	74.84	NR	NR	64.5–70.4	70.97–72.96
2	Ash	2.04	2.11	NR	4.94	4.33	0.87–0.98	0.85	NR	1.17–1.31
3	Protein	3.69	4.80	NR	2.84	2.48	1.44–2.50	0.58	1.9–2.7	1.91–5.83
4	Fats	0.42	0.39	NR	1.00	1.12	0.03–0.95	0.19	1.1–1.7	0.17–0.63
5	Starch	65.41	33.66	NR	NR	NR	NR	26.34	NR	NR
6	Crude fiber (total)	3.68	2.57	2.01–3.23	4.52	3.83	NR	NR	3.0–3.6	0.35–0.54
	Soluble	NR	NR	0.15–1.00	NR	NR	NR	NR	NR	NR
	insoluble	NR	NR	1.35–2.64	NR	NR	NR	NR	NR	NR
7	Total carbohydrate	90.17	90.13	NR	86.72	88.01	23.91–33.45	NR	18.3–26.1	21.10–24.50
9	pH	6.55	6.52	NR	NR		NR	NR	NR	NR
10	Acidity	1.08	0.91	NR	NR		NR	NR	NR	NR
11	Energy (*Kcal/100g.)*	NR	NR	NR	373.97	373.05	NR	344.52	NR	NR

Abbreviation: NR, not reported.

a Samples from Rio de Janeiro, Brazil.

b Sample from Hawaii, range taken from seven varieties (Excel, Jewel, Kona, Makua yam, Regal, South Delite, and UH 71‐5).

c Samples from Ethiopia.

d Sample from three districts (Meatu, Sengerema, and Misungwi) of Lake Zone in Tanzania. (The varieties are Carrot Dar, Japon, Zapallo, and Mafuta.)

e Samples from Brazil.

f Samples from Tanzania (range of Jewel, Karoti Dar, Kabode, Ejumula varieties).

g Range of different varieties (Tripti, Kamala Sundari, Daulatpuri, Misti Alu, Lalkothi, and Kalmegh) from Bangladesh.

h Product from blanched at 100°C for 20 min and hot air oven dried at 65°C for 24 hr.

Moisture is the major component in OFSP, which accounts for >62% and <75% (Endrias et al., 2016; Lyimo, Gimbi, & Kihinga, 2010; Nicanuru, Laswai, & Sila, 2015). Other root and tubers also reported similar moisture contents such as potato (79%), cassava (60%), WFSP (77%), and yam (70%) (USDA, 2018). Usually, roots and tubers are consumed in fresh after boiling or minimal processing with traditional practices. Different researchers reported the various drying techniques for OFSP and converted into flour to the moisture content of <11% (Rodrigues et al.., 2016). The low moisture content is very important for OFSP flour to maintain long shelf life. These similar trends were reported in case of staple grains also, and usually at the time of harvesting, cereals possess moisture contents >22% to 25% and dried to 9%–13% for long‐term storage (Liu, Chen, Wu, & Peng, 2007). The moisture content of OFSP reported variation in different studies; this may be related to the diversity in variety, agroclimatic conditions, agriculture practices employed, etc.

Ash is an inorganic residue in any food substance, which directly denotes the mineral content. The ash values were reported from the range of 1.17%–4.33% (Mohammad et al., 2016), this broad range is obvious, and it happened due to the varietal and agrogeological differences. Also, processes such as peeling and blanching were followed by drying reported on the ash contents (Endrias et al., 2016). The ash contents of OFSP are comparable with other roots and tubers.

Proteins are very important nutrients for structural, functional performers of different biomolecules in human body, and they provide the essential amino acids required for metabolism. The protein in the range of 1.91%–5.83% was reported in OFSP (Mohammad et al., 2016). Usually, roots and tuber are the poor source for protein, which is similar in case of the OFSP. The protein contents of the OFSP are in similar to those of potato (2%), cassava (1.4%), WFSP (1.6%), and yam (1.5). In contrast, the protein compositions of OFSP are very low as compared to the staple food grains, such as maize (9.4%), rice (7.1%), wheat (12.6%), and sorghum (11.6%) (USDA, 2018). So, to combat the Protein Energy Malnutrition (PEM), it is very important to consume the protein‐rich pulses and animal foods among communities where OFSP is considered as second staple (Neumann, Harris, & Rogers, 2002).

Very low concentration (<1%) of the fat was reported in OFSP; usually, this trend is the property of roots and tubers (Lyimo et al., 2010; Mohammad et al., 2016; Rodrigues et al., 2016). The fat concentration of the OFSP is even little better than other roots and tubers such as potato (0.09%), cassava (0.28%), WFSP (0.05%), and yam (0.17%) (USDA, 2018). Usually, staple crops contain less concentration of the oils and fats; this is true in case of rice (0.66%) and wheat (1.54%). The fat concentrations directly influence the energy density of the food, but people with limited energy to avoid certain disease can consume OFSP (Hu, van Dam, & Liu, 2001). However, people in underdeveloped countries in Africa are consuming OFSP more so, and supplementation of the oilseeds and high‐energy foods is highly recommended to certain vulnerable groups (need high energy) (Butte, 2000).

The high starch (65.41%) concentration was reported in OFSP on fresh weight basis (Rodrigues et al., 2016). This is similar to other staple cereal, roots, and tubers. Starch is one of the very important energy sources for the consumers. So, OFSP can consume as the staple crop because of high concentration of carbohydrates (Jobling, 2004).

Crude fiber is one of the nondigestible carbohydrates, which provides the fecal bulkiness, less intestinal transit, role in cholesterol level reduction, and trapping dangerous substance like cancer‐causing agents, and also encourages the growth of natural microbial flora in gut (Dhingra, Michael, Rajput, & Patil, 2012; Sánchez‐Zapata, Viuda‐Martos, Fernández‐LÓpez, & Pérez‐Alvarez, 2015; Satija & Hu, 2012; Slavin, 2013). The total dietary fiber of 3.6% (maximum) was reported in OFSP, but the lower concentrations like 0.35% were reported in different varieties of OFSP (Endrias et al., 2016). These variations may be related to the varietal and agroclimatic differences of the crop. The crude fiber of OFSP is higher than potatoes (2.2%) and cassava (1.8%); however, the comparable concentrations of yam were reported in OFSP. In case of the staple cereals such as maize (7.3%) and wheat (12.2%), they reported high concentration of the fiber than OFSP (USDA, 2018). Usually, cereals contain outer bran layers, which are high source of the crude fibers. The good concentration of crude fiber is an advantage for OFSP consumers. Very limited studies are reported on soluble and insoluble fibers of OFSP, Huang, Tanudjaja, and Lum (1999) reported high concentration of the insoluble dietary fiber (1.35%–2.64%) compared to soluble (0.15%–1.00%), and this is a positive nutritional aspect of the OFSP.

Energy density is a very important property of the staple crops, for example, 344.52–375.05 kcal/100 g in OFSP (Endrias et al., 2016; Nascimento, Dhiego, Cristina, José, & Maria, 2015). The energy of OFSP is comparable with the cereals such as maize (365.2 kcal/100 g) and rice (365 kcal/100 g); in comparison with the other roots and tubers, the energy composition of the OFSP was reported better. Due to this property, the OFSP is one of the choices for staple foods (Prentice & Jebb, 2003).

pH and TSS are very important parameters that influence the taste and overall sensory acceptability of foods. The almost neutral pH (6.52) and TSS (acidity) of 1% (Rodrigues et al., 2016) were reported in the OFSP. It denotes the plain flavor of the OFSP, which is suitable for all age groups as a staple food.

## 4 MINERAL COMPOSITION OF THE OFSP

Minerals are the inorganic components, having very specific and important role in metabolism (Soetan, Olaiya, & Oyewole, 2010). Consumption of optimum concentration of minerals is recommended (Soetan et al., 2010). The mineral compositions of the OFSP from different researchers are tabulated in Table 2.

**Table 2:** Minerals (mg/100 g) in orange‐fleshed Sweet potato reported by different authors from varieties around the world

S. no.	Mineral	RDA mg	Laurie et al. (2012)^a^	Endrias et al. (2016)^b^		Sanoussi et al. (2016)^c^	Ukom et al. (2009)^d^
				Un pealed	Pealed		
1	Calcium	1,000	34–39	47.00	45.54	24.40–29.97	50.20–90.40
2	Magnesium	130	15–37	3.00	UD	23.50–25.40	12.20–30.40
3	Phosphorous	500	28–51	22.11	20.67	42.00–43.67	18.80–27.50
4	Potassium	3,000	191–334	NR	NR	310.67–315.33	138.00–260.00
5	Iron	10	0.73–1.26	15.26	11.45	0.63–0.73	NR
6	Zinc	5	0.51–0.69	1.30	0.93	0.24–0.73	NR
7	Sodium	1,500	NR	NR	NR	30.33–33.10	23.00–59.00

Abbreviation: NR, not reported.

a Range mean was taken from study done by nine OFSP varieties 3 (Khano, Serolane, and Impilo), from South Africa.

b Sample from Ethiopia, UD, undetected, NR, not reported.

c Samples from Benin, range of 4 orange‐fleshed varieties (Carrotti, Loki kpikpa, Dokouin C, and Mansawin).

d Two varieties (CIP‐Tanzania and Ex‐lgbarium) with different conc. of fertilizer.

Calcium plays a major role in muscle function, formation and strengthening of bones, teeth, conducting nerve impulses, blood clotting, and maintaining a normal heartbeat (Zemel, 2009). Humans in the ages of 18–50 require 1,000 mg of calcium per day as recommended daily allowance (RDA). Individuals younger than 18 years need superior concentration (1,300 mg) of Ca for developing bones and teeth (Wosje & Specker, 2000). The calcium content of 24.40–45.54 mg/100 g was reported in OFSP, and this variation in the Ca concentration attributed to varietal and agrogeological conditions (Endrias et al., 2016; Laurie, Jaarsveld, Faber, Philpott, & Labuschagne, 2012; Sanoussi et al., 2016; Ukom, Ojimelukwe, & Okpara, 2009). The Ca content in OFSP was reported as superior to common staple cereals, roots, and tubers. Sorghum (28 mg/100 g), maize (7 mg/100 g), rice (28 mg/100 g), wheat (29 mg/100 g), potato (12 mg/100 g), cassava (6 mg/100 g), WFSP (30 mg/100 g), and yam (17 mg/100 g) are reported to have superior concentration of Ca than common staples (USDA, 2018). In all the common roots and tubers, high concentration of the Ca, high amount of OFSP has to consume, or the food rich in Ca (animal‐based food) has to incorporate into the staple diets to achieve RDA of Ca was reported in OFSP.

Magnesium is one of the six important key macrominerals and essential mineral in >300 metabolic functions and possesses role in strong bones, appropriate muscle tasks, optimal blood pressure, and appropriate cardiac tempo (Saris, Mervaala, Karppanen, Khawaja, & Lewenstam, 2000). Sales and Pedrosa (2006) reported the DNA synthesis and stability depend on magnesium. The RDA of Mg to men and women is 420 and 320 mg, respectively. The Mg concentration of 3–37 mg/100 g was reported in OFSP, and this variation was attributed to the varietal and agroclimatic conditions (Endrias et al., 2016; Laurie et al., 2012; Sanoussi et al., 2016; Ukom et al., 2009). Staple cereals are good source of the Mg than OFSP and other roots and tubers. Maize and wheat contain high concentration of the Mg (125 mg/100 g), but rice (25 mg/100 g) and other tuber crops such as potato (23 mg/100 g), cassava (21 mg/100 g), WFSP (31 mg/100 g), and yam (21 mg/100 g) are similar to OFSP (USDA, 2018). The Mg‐rich food supplementation is highly recommended in the consumers of OFSP to meet RDA of magnesium.

Phosphorus is a necessary mineral in human body after calcium and possesses a pivotal role in abundant metabolic process, including energy metabolism and bone mineralization, and DNA and RNA framework (Karp, Vaihia, Kärkkäinen, Niemistö, & Lamberg‐Allardt, 2007). The RDA of 700 mg phosphorus is for healthy adults, and phosphorous of 15–51 mg/100 g was reported in OFSP, which is almost equal to the other roots and tubers such as potato (57 mg/100 g), cassava (27 mg/100 g), WFSP (47 mg/100 g), and yam (55 mg/100 g) (Endrias et al., 2016; Lyimo et al., 2010; Nicanuru et al., 2015). In contrast, staple grains contain high concentration of the phosphorous, maize, rice, and wheat are reported to have 210 mg/100 g, 115 mg/100 g, and 288 mg/100 g of phosphorous, respectively (USDA, 2018). The consumption of whole grains is recommended achieving RDA of phosphorous along with the OFSP.

Potassium along with Na and Ca regulates the fluid balance, maintains normal heart rhythm (Khaw & Barrett‐Connor, 1987), and is accountable for nerve signals and muscle functions. Potassium in the range of 138–334 mg/100 g was reported in OFSP (Endrias et al., 2016; Lyimo et al., 2010; Nicanuru et al., 2015), and these amounts are better than rice (115 mg/100 g) and maize (287 mg/100 g), but wheat (363 mg/100 g) contains similar amount of K with OFSP (USDA, 2018). In case of roots and tubers, cassava (271 mg/100 g) contains lesser concentration of potassium than OFSP, but potato (421 mg/100 g) and yam (816 mg/100 g) contain higher amount of K, and among all, yam is rich source of potassium. Children older than 13 years and adults RDA of K are 4,700 mg, but lactating women require 5,100 mg. The roots and tubers are good source of K, and OFSP is moderately providing the RDA of potassium for consumers (Constán‐Aguilar et al., 2014).

About 6% of iron in humans are present in certain proteins, which are crucial for respiration and energy metabolism process and implicated in the amalgamation of collagen and certain neurotransmitters (Bashiri, Burstein, Sheiner, & Mazor, 2003). About quarter of the Fe in humans is deposited as ferritin, located in cells, and circulated in the blood circulation system. Iron deficiency is known as erythropoiesis, and worst Fe deficiency leads to anemia (Abbaspour, Hurrell, & Kelishadi, 2014). The RDA of iron is 1.8 mg in adults, and merely 10%–30% of the Fe in diet is bioavailable (Endrias et al., 2016; Lyimo et al., 2010; Nicanuru et al., 2015). OFSP was reported to be 0.63–15.26 mg/100 g of iron, whereas maize (2.71 mg/100 g), rice (0.8 mg/100 g), wheat (3.19 mg/100 g), potato (0.78 mg/100 g), cassava (0.27 mg/100 g), WFSP (0.61 mg/100 g), and yam (0.54 mg/100 g) reported to be lesser than the OFSP (USDA, 2018). So, OFSP is a good source for providing the RDA of iron.

Zn plays an important role in body where deficiency symptoms are shown in many ways (Powell, 2000). Antinutritional factors are prime known inhibitor of zinc, which abundantly present in cereals and grains (Janet, 2003). Zinc is required for good immune system function, cell growth, wound healing, and insulin function (Chausmer, 1998). OFSP was reported to be 0.24–0.93 mg/100 g of zinc, but staple food grains such as maize (2.21 mg/100 g), rice (1.09 mg/100 g), and wheat (2.65 mg/100 g) have high concentration of the Zn, whereas potatoes (0.29 mg/100 g), cassava (0.34 mg/100 g), WFSP (0.3 mg/100 g), and yam (0.24 mg/100 g) contain the zinc in the range of OFSP (Endrias et al., 2016; Lyimo et al., 2010; Nicanuru et al., 2015). RDA of Zn varied depends on the age, 14 years old and above need 11 mg/day, and women in age 19 and above need 8 mg/day (Lutter & Rivera, 2003), whereas pregnant and lactating women need 13 and 14 mg/day, respectively. OFSP provides very small quantities of the Zn, but the bioavailability is more comparative to the cereals and grains because of no or less antinutritional factors (phytate) (Wanasundera & Ravindran, 1994).

Human body acquires sodium from food and drink, and losses through sweat and urine; kidneys play a crucial role in sodium‐level adjustments (Reynolds, Padfield, & Seckl, 2006). OFSP was reported to be 23–59 mg/100 mg, staples foods such as maize (35 mg/100 mg) contain the Na within the range of OFSP; however, rice (5 mg/100 mg), wheat (2 mg/100 mg), potato (6 mg/100 mg), cassava (14 mg/100 mg), and yam (9 mg/100 mg) contain less concentrations (Endrias et al., 2016; Lyimo et al., 2010; Nicanuru et al., 2015). RDA of sodium is 1,500 mg; less concentrations of the sodium in food source may not have any health problem, because the addition of sodium in form of table salt is a common practice in human food preparation for sake of taste.

## 5 CAROTENES IN OFSP

Carotenoids are natural pigments; more than 750 types are present in divergent algae and photosynthetic bacteria. These carotenoids are accountable for yellow, orange, and red colors in different plant products (fruits, vegetables, and flowers), distinct aromatic scents, and flavors (Namitha & Negi, 2010).

In plants, carotenoids are located in chromoplasts and chloroplasts, and possess pivotal roles in photosynthetic guard from photo‐oxidative injuries (Carol & Kuntz, 2001). Carotenoids are metabolized in the plastid by methylerythritol‐4‐phosphate (MEP) pathway (Phillips, León, Boronat, & Rodríguez‐Concepción, 2008). This biopathway is composed of two branches behind lycopene to produce AC and BC by lycopene ε‐cyclase (LCYE) and lycopene β‐cyclase (LCYB) (Fraser & Bramley, 2004).

### 5.1 β‐Cryptoxanthin

β‐Cryptoxanthin (BCX) is the most common dietary carotenoids found in the fruits and vegetables, and it is a PVAC along with AC and BC (Berni, Chitchumroonchokchai, Canniatti‐Brazaca, De Moura, & Failla, 2015; Rao & Rao, 2007). 24 µg of BCX in food will metabolize to 1 μg of retinol in human body (Weber & Grune, 2012). Medical studies reported the risk of pulmonary cancer was estimated to reduce 20% with consumption of BCX (Lian, Hu, Russell, & Wang, 2006; Yuan, Ross, Chu, Gao, & Yu, 2001). Research reports also concluded that superior consumption of carotenoids (BCX and lycopene) possesses optimistic connection with a reduced mouth cancer risk (Terry, Lagergren, Hansen, Wolk, & Nyrén, 2001). Researchers proved that high concentration of NPVAC (lutein, zeaxanthin and lycopene) to VAC (AC, BC, BCX) in plasma has positive role in reducing diabetic retinopathy in type 2 diabetes mellitus patients (Brazionis, Rowley, Itsiopoulos, & O'dea, 2009). Lower concentrations of high‐density lipid (HDL) concentration in body was found with BCX intake by food, and also recent experimental study reported the BCX positive correlation with osteoporosis prevention (Gammone, Riccioni, & D'Orazio, 2015).

Limited studies were reported on concentration of BCX in OFSP as 21.2 (µg/g db) (Kim et al., 2015). So, consumption of OFSP with BCX concentration will contribute to the 0.5 µg of VA by 1 g consumption. So, 100 g of OFSP will provide 50 µg of VA, and it contributes to the VA RDA requirements with BCX, AC, and BC. Some foods such as pumpkin, papaya, oranges, carrots, and yellow corn are rich sources of BCX. The comparable BCX concentrations in agreement with other foods is reported in OFSP (Breithaupt & Bamedi, 2001). The composition of different carotenoids and their concentrations in OFSP are reported in Table 3.

**Table 3:** Composition of different carotenoids and vitamins in orange‐fleshed sweet potato (OFSP) reported by different authors

Reference	α‐Carotene	β‐Carotene	Total carotene	Anthocyanin (mg/g fw)	Ascorbic acid	β‐Cryptoxanthin	α‐Tocopherol	Lutein	Zeaxanthin
Stinco et al. (2014)^a^	13.11 (µg/g db)	48.66 (µg/g db)	61.77 (µg/g db)	NR	NR	NR	NR	NR	NR
Teow et al. (2007)^b^	NR	44.9–226 (µg/g fw)	NR	17–38 (µg/g fw)	NR	NR	NR	NR	NR
Shih et al. (2009)^c^	NR	34.6–83.3 (µg/g db)	NR	4.0–8.5 (µg/g db)	NR	NR	NR	NR	NR
Tomlins et al. (2012)^d^	NR	20–364 (µg/g db)	41.7–251 (µg/g db)	NR	NR	NR	NR	NR	NR
Grace et al. (2014)^e^	NR	NR	NR	ND	870 (µg/g db)	NR	NR	NR	NR
Islam et al. (2016)^f^	NR	NR	19.31–61.94 (µg/g fw)	NR	NR	NR	NR	NR	NR
van Jaarsveld et al. (2006)^g^	NR	132–194 (µg/gfw)	NR	NR	NR	NR	NR	NR	NR
Kim et al. (2015)^h^	NR	NR	570 (µg/g db)	NR	NR	21.2 (µg/g db)	NR	NR	NR
Lebot et al. (2016)^i^	NR	NR	NR	0–1558 (mean 243 [AU, area units])	NR	NR	NR	NR	NR
Wu et al. (2008)^j^	NR	6.2–231 (µg/gfw)	NR	NR	NR	NR	NR	NR	NR
Tang et al. (2015)^k^	NR	NR	NR	2.9 (µ/g)	NR	NR	NR	NR	NR
Brown et al. (1993)^l^	NR	NR	NR	NR	NR	NR	NR	120–148 (µg/g fw)	242–2,055 (µg/g fw)
Donado‐Pestana et al. (2012)^m^	NR	NR	NR	NR	NR	NR	NR	1–4 (µg/g db)	1–2 (µg/g db)
(Mohammad et al. (2016)^n^	NR	NR	5.5–72.4 (µg/g fw)	NR	NR	NR	NR	NR	NR
Huang et al. (1999)^o^	1–15 (µg/g fw)	67–131 (µg/g fw)	NR	NR	NR	NR	NR	NR	NR
Oki et al. (2006)^p^	NR	85.36–177.16 (µg/g fw)	NR	NR	NR	NR	2.61–8.48 (µg/g fw)	NR	NR
Tumuhimbise et al. (2009)^q^	NR	67.33–315.71 (µg/g db)	NR	NR	NR	NR		NR	NR
Vimala, Nambisan, and Hariprakash (2011)^r^	NR	0.96–13.6 (µg/g fw)	15–155 (µg/g fw)	NR	NR	NR		NR	NR

Abbreviation: NR, not reported.

a Values from (Rubina, Diane, California Organic, Evangeline, Darby, Beauregard varieties) from the United States and Israel.

b Ranges from Beauregard (Beau), Hernandez (Hern), Covington (Covin), 11–5, 11–20,13–14,13–15 from the United States.

c Tainong 66, from Taiwan.

d Range from different varieties (Ejumula, Kakamega, SPK004/1, SPK004/6/6, and SPK004/ 1/1) from Uganda.

e Covington variety fresh sample from the United States.

f Ranges from Kamalasundari, BARI SP 4, BARI SP 5, BARI SP 6.

g Raw Resisto, from the same harvest batch.

h Sinhwangmi, from Korea.

i OFSP from France.

j Samples procured from Shandong, Sichuan, Jiangsu, Fujian, Sichuan places, and range was taken from the varieties of Ji03314, Ji03468, S01009, S01056, Xushu 22‐5, xushu, Yanshu, 200730 from China.

k Guineng 05‐6 (orange), cyanidin‐3‐glucoside (cyE).

l Ranges taken from D6.11, 90B10.2, 90B 10.20, 90B 10.35 varieties.

m The values that are taken range from varieties of CNPH1007, CNPH1194, CNPH1202, CNPH 1205 from Brazil.

n BARI SP1 to 9 varieties from Bangladesh.

o Range taken from varieties Excel, Jewel, Kona B, Makua yam, Regal, South Delite, UH 71‐5 from Hawaii.

p Range taken from Kyushu, 144, 1243, 241; sunnyred, Hamakomachi, J‐Red, Ayakomachi from Japan.

q Ejumula, SPK004/6/6, SPK004/6, SPK004, SPK0041 from Uganda.

r Range taken from the varieties KS‐7, ST‐14‐1, ST‐14‐16, ST‐14‐34, ST‐14‐49, ST‐14‐53, ST‐14‐6, ST14‐9, SV‐3‐17, SV‐3‐22, from India.

### 5.2 Lutein and zeaxanthin

Lutein (β,ε‐carotene‐3′,‐diol) is naturally present with zeaxanthin (β, β‐carotene‐3,3′‐diol) as stereoisomer and available in large quantities in leafy vegetables (Perry, Rasmussen, & Johnson, 2009). Lutein and zeaxanthin are structured with 40 carbon atoms and named as tetraterpenoids, and they are synthesized only in plant kingdom (Shegokar & Mitri, 2012). Lutein and zeaxanthin belong to xanthophyll family and do not possess any VA activity (Abdel‐Aal, Akhtar, Zaheer, & Ali, 2013). The yellow colors of these two xanthophylls are connected to their structural properties and responsible for orange to yellow color of different vegetables and animal products (egg yolk, animal fat). The animal retina is exceptional and contains a yellow colored region positioned in its ocular center known as “macula lutea” (Kalariya, Ramana, & vanKuijk, 2012). This yellow color attributed to the accumulation of lutein, zeaxanthin, and meso‐zeaxanthin, and they are very important in eye well‐being (Kijlstra, Tian, Kelly, & Berendschot, 2012; Nwachukwu, Udenigwe, & Aluko, 2016). Xanthophylls play a critical role in the defense of skin against sunlight (Stahl & Sies, 2007). Lutein and zeaxanthin protect eye from high‐energy blue light, and inhibit and repair the photoinduced oxidative damage (Bian et al., 2012).

Limited reports were available on the concentration of the lutein and zeaxanthin in OFSP. Brown, Edwards, Yang, and Dean (1993) reported the 120–148 (µg/g fw) and 242–2,055 (µg/g fw) of lutein and zeaxanthin respectively, whereas Donado‐Pestana et al. (2012) reported 1–4 (µg/g db) and 1–2 (µg/g db) of lutein and zeaxanthin in OFSP, respectively. The orange color of the OFSP is devoted to the presence of this lutein and zeaxanthins. The differences in the reported concentrations of the two reports may be attributed to the variety‐related issues. Researchers reported the intensity variations of orange color among the cultivars in different countries, and this was directly linked to the concentration of lutein + zeaxanthin. However, the lutein + zeaxanthin by Brown et al. (1993) reported high concentration in OFSP, which is comparable with the other leafy vegetables. Vegetables such as baby carrots, peaches, corn, papaya, and oranges possess <1 mg of lutein + zeaxanthin in 100 g of fresh weight (Holden et al., 1999). Ziegler et al. (2015) reported the limited quantity of lutein esters in cereals; in contrast, xanthophylls are rich in fruits and vegetables such as collards, capsicum, yellow corn, and spinach (Sajilata, Singhal, & Kamat, 2008). Consumption of OFSP also provides the required xanthophylls to the consumers. Recent studies reported that these two xanthophylls possess very important role in different disease management such as skin damages, reduce the chance of pancreatic cancer, reducing the CHD, and increase in overall antioxidant activity (Gordon, 1996; Mares‐Perlman, Millen, Ficek, & Hankinson, 2002).

### 5.3 α, β‐Carotenes (AC and BC)

AC and BC are prime bases for VA; they metabolize to retinol in humans. Fruits and vegetables with orange, yellow, and green color are rich source of these two carotenoids (Saini, Nile, & Park, 2015). The VA conversion efficacy of BC is double in comparison with AC, one portion of BC yields two portions of retinol, but AC yields only half portion (Thurnham, 2007). Due to the large concentrations of the BC shares with AC in plant sources, its medicinal values are not well established (Murakoshi et al., 1992).

Variations in polar groups determine the metabolic roles of carotenoids (Britton, 2008). Carotenoids are very significant for AA, intercellular signal transmission and body defense mechanisms (Chew & Park, 2004; Edge, Mc Garvey, & Truscott, 1997; Kumar, Singh, & Ekavali, 2015), lung cancer (Ziegler, 1989), and coronary health and vision problems (Meyers et al., 2013; Sharoni et al., 2012). Shortage of carotenoid consequences with broader medical symptoms related to the eye and vision (Sommer, 2008) weaken innate, adaptive immunity (Stephensen, 2001). Levy et al. (1995) reported that BC is very effective in the suppression of malignant tumor cells.

The OFSP is considered as the good source of the carotenoids, and many researches are recommended to use the OFSP to combat the problems of VAD in developing countries. Stinco, Benítez‐González, Hernanz, Vicario, and Meléndez‐Martínez (2014) reported 13.11 (µg/g db), and Huang et al. (1999) reported 1–15 (µg/g fw) of AC in OFSP. Many researchers reported the BC concentrations are higher than AC in fresh and dry basis. Among the all, Tomlins, Owori, Bechoff, Menya, and Westby (2012) reported the highest range (20–364 (µg/g db)) of the BC in OFSP from different varieties grown in Uganda, whereas Teow et al. (2007) reported the 44.9–226 (µg/g fw) of the BC in fresh base from US varieties of OFSP. Overall, the presence of high concentration of BC in the OFSP is a good sign as previously discussed as BC yields high amount of the PVA. Both the carotenes are very high in OFSP compared to the common consuming yellow to orange vegetables and fruits. As reported by the Gul et al. (2015) the carotenoid concentration in different foods, such as carrot (43.5–88.4 µg/g), mango (10.9–12.1 µg/g), and tomato (2.17–2.83 µg/g), contain the lower concentration of the BC than the OFSP. In case of the total carotenoids, 570 µg/g (db) of the carotenoids are reported by Kim et al. (2015) in OFSP from Korea. These concentrations are much higher than any other fruits and vegetables.

### 5.4 Carotenoid isomers in OFSP

Carotenoids naturally exist in different isomers; obviously, the majority of the carotenoids happen in *trans*‐isomers in vegetative source. Nevertheless, rise in *cis*‐isomers attributed to food processing conditions (Schieber & Carle, 2005). Many authors, Mertz, Brat, Caris‐Veyrat, and Gunata (2010), Shi et al. (2010), and Zepka and Mercadante (2009) reported the different isomers in the dietary carotenoids, whereas some researchers are focused on geometric isomerization of carotenoids (Niedzwiedzki, Enriquez, Lafountain, & Frank, 2010; Qiu, Chen, & Li, 2009).

Food processing conditions such as application of heat, exposure of radiation, and innate structural variations are responsible for variation in carotenoid isomer formations (Chwartz, 2002; De Rigal, Gauillard, & Richard‐Forget, 2000; Kumar et al., 2015; Parker, 1996).

Carotenoids with seven and more double bonds (conjugated) are identified for good AA (Borsarelli & Mercadante, 2009). *Trans*‐BC is highly sensitive to heat and light and isomerizes to *cis*‐isomers when exposed; for instance, Vásquez‐Caicedo, Schilling, Carle, and Neidhart (2007) reported the processing of fruits leads to *cis*‐*trans* isomer formation and this processes is very particular in case of 13‐*cis*‐BC formations (Lozano‐Alejo, Carrillo, Pixley, & Palacios‐Rojas, 2007). 9‐*cis*‐BC in various foods was reported when exposed to light (Lozano‐Alejo et al., 2007; Schieber & Carle, 2005), while 13‐*cis*‐AC and BC isomers are identified in extended storage durations (Tang & Chen, 2000).

Chen and Huang (1998) reported that the reflux heating converts BC to all‐*trans*‐BC and 13‐*cis*‐BC. Pasteurized and sterilized carrot juice reported the presence of high concentration of 13‐*cis*‐BC, while 9‐*cis*‐BC was reported in blanched conditions. Moreover, 9‐*cis*‐ and 13‐*cis*‐BC were reported to produce independently from *cis* bases due to the nonenzymatic actions of all‐*trans* forms (Marx, Stuparic, Schieber, & Carle, 2003). In contrast, heating and exposure to air of *cis*‐BC found no difference in all‐*trans*‐isomer (Qiu et al., 2009); however, studies conclude that BC undergoes to the isomerization rather degradation when exposed to adverse condition. Recent studies reported that all‐*trans* forms contain higher biological availability than *cis‐*counterpart, while BC and β‐apo‐12′‐carotenal have the highest bioconversion rate (Castenmiller & West, 1998).

In case of OFSP, Islam, Nusrat, Begum, and Ahsan (2016) reported *cis*‐BC of 3.5–23.44 µg/g (fw), Liu, Lin, and Yang (2009) reported di‐*cis*‐BC 1.5–5.7 µg/g (fw), whereas Donado‐Pestana et al. (2012) reported 70–113 µg/g (db) of 5,6 *epoxy*‐BC. Berni et al. (2015) reported 13‐ or 13′‐*cis*‐AC of 3.8–9.0 µg/g (fw), 15‐ or 15′‐*cis*‐BC 1.9–3.5 µg/g (fw), but 0.004–0.39 µg/g (fw). Comparatively other isomers of carotenoids, 13‐ or 13′‐*cis*‐BC concentrations in OFSP, were reported to be 4.7–94 µg/g (db) from the studies reported by Bengtsson, Namutebi, Alminger, and Svanberg (2008), Donado‐Pestana et al. (2012), Kim et al. (2015), and Tumuhimbise, Namutebi, and Muyonga (2009). 1.14–4.7 µg/g (fw) in range of the 13‐ or 13′‐*cis*‐BC in OFSP was reported by Berni et al. (2015) and Liu et al. (2009). The concentration of 9‐ or 9′‐*cis*‐BC was identified in the range of 0.006–9.8 µg/g (fw) in the studies of Berni et al. (2015) and Liu et al. (2009). In case of dry OFSP, samples were reported to be 7.4–618 (µg/g) range of 9‐ or 9′‐*cis*‐ BC by Donado‐Pestana et al. (2012) and Kim et al. (2015). All‐*trans*‐AC was reported to be 1.2–2.7 µg/g (fw) by only Liu et al. (2009), whereas All‐*trans*‐BC concentration was reported to be in the range of 0.01–1285 µg/g (fw) by studies of Berni et al. (2015), Donado‐Pestana et al. (2012), Kim et al. (2015), Liu et al. (2009), and Tumuhimbise et al. (2009), whereas in dry base, it was shown that all‐*trans*‐BC was reported to be 0.01–114.4 µg/g by, Berni et al. (2015), Islam et al. (2016), and Liu et al. (2009). Liu et al. (2009) reported total *cis‐*isomers of AC and total *cis*‐isomers of BC of 3.8–0.9.0 and 14.4–33.1 µg/g (fw), respectively. The concentrations of carotenoid isomers in OFSP from different locations, and authors are presented in Table 4.

**Table 4:** Concentrations of carotenoid isomers in orange‐fleshed sweet potatoes reported by different authors

Isomers of carotene	Liu et al. (2009)^a^ (µg/g fw)	Islam et al. (2016)^b^ (µg/g fw)	Bengtsson et al. (2008)^c^ (µg/g db)	Kim et al. (2015)^d^ (µg/g db)	Berni et al. (2015)^e^ (µg/g fw)	Donado‐Pestana et al. (2012)^f^ (µg/g db)	Tumuhimbise et al. (2009)^g^ (µg/g db)
*cis*‐β‐Carotene	4.3–9.3	3.5–23.44	NR	NR	NR	NR	NR
di‐*cis*‐β‐Carotene	1.5–5.7	NR	NR	NR	NR	NR	NR
5,6 epoxy‐β‐ carotene	NR	NR	NR	NR	NR	70–113	NR
13‐ or 13′‐*cis*‐α‐Carotene	3.8–9.0	NR	NR	NR	NR	NR	NR
15‐ or 15′‐*cis*‐ β‐Carotene	1.9–3.5	NR	NR	NR	0.004–0.39	NR	NR
13‐ or 13′‐cis‐ β‐Carotene	3.0–4.7	NR	4.7–15.9	11.6	1.14–2.30	88–94	0.75–1.22
9‐ or 9′‐*cis*‐β‐Carotene	7–9.8	NR	NR	7.4	0.006–2.82	55–61	NR
All‐*trans*‐α‐Carotene	1.2–2.7	NR	NR	NR	NR		NR
All‐*trans*‐β‐Carotene	2.7–37.6	76.59–96.49	108.1–314.5	530	0.01–114.4	791–1285	66.79–314.48
Total *cis*‐isomers of α‐carotene	3.8–0.9.0	NR	NR	NR	0.11–0.27	NR	NR
Total *cis*‐isomers of β‐carotene	14.4–33.1	NR	NR	NR	NR	NR	NR

a Tainung 66 (with orange flesh) range from harvested from January to October, from Taiwan.

b Ranges from Kamalasundari, BARI SP 4, BARI SP 5, BARI SP 6.

c SPK004/1, SPK004/6, SPK004, Sowola 6/94/9, SPK 004/1, SPK 004/6/6, Ejumula.

d Sinhwangmi, from Korea.

e Brazlandia, Amelia, Beauregard.

f The values that are taken range from varieties of CNPH1007, CNPH1194, CNPH1202, CNPH1205 from Brazil.

g Ejumula, SPK004/6/6, SPK004/6, SPK004, SPK0041 from Uganda.

## 6 α‐TOCOPHEROL

Vitamin E represents eight isomers of fat‐soluble compounds (α‐, β‐, γ‐,δ‐tocopherol and tocotrienol) metabolized in plants (Bjorneboe, Bjorneboe, & Drevon, 1990; Helmut, Wilhelem, & Alfred, 1992; Netscher, 2007), available in various concentrations in fat‐rich foods (edible oils and seeds) (Reboul et al., 2006; Yanishlieva & Marinova, 2001). α‐Tocopherol (Pryor, 2000) is appreciated for cytoprotective nature, anti‐inflammatory, and liver protection (Galli et al., 2017), and also considered as potential AA and immune system booster, to fight against viruses and pathogenic bacteria (Traber & Atkinson, 2007). Tocopherol also helps to synthesize new red blood cells and widens blood vessels (Evelyne et al., 1994), potentially lowering risk of developing blood clots (Krem & Cera, 2002). Vit. E RDA is between 3,000 and 15,000 µg in different countries, and the variation reported depends on the age (Jiang, Christen, Mark, & Bruce, 2001). Limited studies were reported on the concentration of the α‐tocopherol (Table 3) in OFSP, and the range of 2.61–8.48 (µg/g fw) was reported by Oki et al. (2006). Consumption of 100–200 gm of OFSP requires achieving around 20%–25% RDA of Vit. E. However, the OFSP is not considered as the best source of the α‐tocopherol because less concentration of fats was reported in OFSP. So, supplementation of Vit. E‐rich foods such as oilseeds is very important for the consumers of OFSP as second staple.

## 7 ASCORBIC ACID

Vitamin C naturally presents in two isomers as ascorbic and dehydroascorbic acid, and they are metabolites in various plants, animals, and fungi (Drouin, Godin, & Page, 2011). The biological activity of ascorbic acid depends on redox state, acting as cofactor for eight human enzymes and different antioxidants (Padayatty & Levine, 2016). Vit. C is known as one of the safest and most effective nutrients, and involves in immune system functions (Padayatty et al., 2003), cardiovascular disease (Simon, 1992), prenatal health problems, eye disease, and skin wrinkling (Vitamin C & Eye Health, 2007).

Vitamin C is extremely essential in synthesis of collagen (Varani et al., 2000), carnitine (Johnston, Solomon, & Corte, 1996), and neurotransmitters (Lee, Lee, Jung, & Lee, 2001), and in the formation and maintenance of bone material (Wang, Villa, Marcus, & Kelsey, 1997), to fight against the oxidative stress (Bendich & Langseth, 1995). Adult men and women require 90 and 75 mg of the Vit. C as the RDA, respectively (Naidu, 2003). Grace et al. (2014) reported (Table 3) Vit. C concentration in OFSP as 870 µg/g (db) of ascorbic acid, which is very less than the different fruits and vegetables.

## 8 ANTHOCYANINS

Anthocyanins are very essential pigments in vascular plants, responsible for glittery orange, pink, red, violet, and blue colors in plants (Glover & Cathie, 2012); they are safe and effortless to apply in aqueous media as natural water‐soluble dyes (Wrolstad, 2000). They naturally occur as glycosides, and anthocyanidins are bound with different sugar groups (Kong, Chia, Goh, Chia, & Brouillard, 2003). Anthocyanidins are highly appreciated due to the antioxidant, anti‐inflammatory, anticarcinogenic activities, protection against heart disease (Bowen‐Forbes, Zhang, & Nair, 2010; Miguel, 2011), and diabetes reduction (Sancho & Pastore, 2012).

Anthocyanin consumption in worldwide reported in high variation depends on the diet composition and eating patterns of the individuals. Studies identified the deviations in the anthocyanin intake in different countries such as the United States (12.5 mg/day) (Wu et al., 2006), the Netherlands (19.8 mg/day), Italy (64.9 mg/day), and Spain (18.4 mg/day) (Zamora‐Ros et al., 2013). In case of OFSP, different authors reported the concentration of ACN. Teow et al. (2007) reported 17–38 (µg/g fw), Shih, Kuo, and Chiang (2009) reported 4.0–8.5 (µg/g db), and Tang et al. (2015) reported 2.9 (µCyE/g) anthocyanins in OFSP (Table 3). The variation in the concentrations reported in OFSP by different researchers may be due to the agrogeological conditions. However, the OFSP is not the good source of the anthocyanin compared to the other fruits and vegetables.

## 9 PHENOLIC ACIDS

Phenolic acids, especially caffeic (CA), ferulic (FA), sinapic (SA), and *p*‐coumaric acids (PCAs) are present in good quantities in variety of fruits and vegetables (Naczk & Shahidi, 2006). Chlorogenic acid (CGA) is an important hydroxycinnamic acid in food with high AA (Acosta‐Estrada, Gutiérrez‐Uribe, & Serna‐Saldívar, 2014), and its bioavailability depends on absorption and metabolism in human alimentary tract. Gonthier, Verny, Besson, Rémésy, and Scalbert (2003) identified the resultant aromatic products (*m*‐coumaric acid and derivatives of phenylpropionic and benzoic acids) by gut microbial action on CGA. These resultant metabolites are very important to activity of polyphenols poorly absorbed in the alimentary tract.

CGA and CA are reported for good AA, inhibition of N‐nitroso compounds (Kono, Shibata, Kodama, & Sawa, 1995), protection of DNA from damage (Cinkilic et al., 2013), cardiovascular disease (Mirella et al.., 1995), antimutagenic properties (Yamada & Tomita, 1996), scavenge of reactive oxygen species (Sato et al., 2011), and inhibition of 8‐dehydroxy‐deoxyguanosine (involved in DNA breakage) (Kasai, Fukada, Yamaizumi, Sugie, & Mori, 2000). CGA reported as novel insulin sensitizer limits the blood glucose concentrations by inhibiting G‐6‐Pase enzyme action (Meng, Cao, Feng, Peng, & Hu, 2013).

The most common form of CGA is 5‐caffeoylquinic acid (5‐CQA). Three major subclasses of CGAs are caffeoylquinic (CQA), feruloylquinic (FQA), and dicaffeoylquinic (diCQA) acids. The major CGA isomers are 3‐, 4‐, and 5‐CQA; 3‐,4‐; 3, 5‐, and 4,5‐CQA; 3,4‐; 3,5‐ and 4,5‐diCQA; 3‐,4‐, and 5‐FQA; 3‐,4‐, and 5‐*p*‐coumaroylquinic acids (Catherine, Nicholas, & George, 2014; Clifford, 2000).

Highest concentration of the CA was reported to be 113.4–490 (µg/g db) by Grace et al. (2014) and Padda and Picha (2008) in OFSP. Lebot, Michalet, and Legendre (2016) reported CA concentration of 0–16,000 µg/g (fw) with mean of 610 (µg/g fw). Other authors reported CA concentration of 4.7–50 (µg/g db) (Grace et al., 2014; Padda & Picha, 2008), but they were not reported FA. In case of the total diCQA, researchers reported 900 (µg/g db) (Grace et al. (2014)). In case of fresh weight base, researchers reported 0–27,791 (AU) of total diCQA with mean of 11,020 (AU). The isomers of diCQA, 4,5‐diCQA were reported to be 9.2–32.4 (µg/g db) (Padda & Picha, 2008), but Lebot et al. (2016) reported (0–1030) mean of 310 (µg/g fw) (e.g., 3,4 diCQA). Other isomer 3,5‐diCQA is reported in dry weight base to be from 56.3 to 300 (µg/g db) as reported by Grace et al. (2014) and Padda and Picha (2008), whereas in wet base weight (0–430) with mean of 170 (µg/g fw), it was reported by Lebot et al. (2016). 3,4‐diCQA was reported to be in the range of 1.9–20 (µg/g db) by Grace et al. (2014) and Padda and Picha (2008). Comparative to the other fruits and vegetables, the low concentration of the phenolic acids was reported in OFSP. Researchers reported wide variation in the total phenolic acid concentration in OFSP in different varieties and grown in wider geographical locations. The concentration of individual phenolic acids reported by the different phenolic acids is tabulated in Table 5.

**Table 5:** Individual phenolic acid contents in orange‐fleshed sweet potato of different varieties

Author	Chlorogenic acid	Caffeic acid	4,5‐Dicaffeoylquinic acid	3,5‐Dicaffeoylquinic acid	3,4‐Dicaffeoylquinic acid	Total dicaffeoylquinic acid
Padda and Picha (2008)^a^ (µg/g db)	113.4–357.8	4.7–13.4	9.2–32.4	56.3–268.4	1.9–12.3	NR
Grace et al. (2014)^b^ (µg/g db)	490	50	20	300	20	900
Lebot et al. (2016)^c^ (µg/g fw)	(0–1,600) Mean 610	NR	(0–1030) Mean 310 (include 3,4 diCQA)	(0–430) Mean 170	NR	(0−27,791 AU) Mean 11,020 (AU)

Abbreviations: CafA, caffeic acid; ChlA, chlorogenic acid; diCQA, dicaffeoylquinic acid.

a OFSP values (Rubina, Diane, California Organic, Evangeline, Darby, Beauregard varieties) from the United States and Israel.

b Covington variety fresh samples from the United States.

c OFSP from France (*n* = 64).

The total phenolic acid concentration of 1.8–310.7 mg/100 g was reported by Donado‐Pestana et al. (2012), Grace et al. (2014), Padda and Picha (2008), and Rumbaoa et al. (2009). Different scientists reported the 0.130–136.05 mg in 100 g of the phenolic acids (Mohammad et al., 2016; Rumbaoa et al., 2009; Teow et al., 2007). CGA accumulation identified high levels in potatoes (2004), and Andre et al. (2007) and Brown (2005) reported the agriculture trails to boost up the phenolic acids and/or carotenoids. Eichhorn and Winterhalter (2005) reported that potato tubers are rich source of flavonoids and CAN available in the form of acylated glycosides located in vacuoles (Kosieradzka, Borucki, Matysiak‐Kata, Szopa, & Sawosz, 2004). High positive correlations were found between total phenolic acids, and anthocyanin presence with antioxidant capacity was reported by many studies (Moyer, Hummer, Finn, Frei, Ronald & Wrolstad, 2002; Pantelidis, Vasilakakis, Manganaris, & Diamantidis, 2007; Prior et al., 1998; Tsai, McIntosh, Pearce, Camden, & Jordan, 2002; Velioglu, Mazza, Gao, & Oomah, 1998).

## 10 ANTIOXIDANT ACTIVITY OF OFSP

Oxidation response leads to the many problems in humans, and they are connected with pathophysiology problems of many diseases (Blokhina, Virolainen, & Fagerstedt, 2003; Lipinski, 2001), which includes inflammation (Halliwell, 2006), cancer (Reuter, Gupta, Chaturvedi, & Aggarwal, 2010), atherosclerosis, and aging (Finkel & Holbrook, 2000). Antioxidants occur naturally or chemically synthesizing, and foods from the higher plants are rich source of natural antioxidants (tocopherols and polyphenols) (Imark, Kneubühl, & Bodmer, 2000). Naturally occurring antioxidant isolation and application in food and pharmaceutical industries are already existed (Suhaj, 2006).

In case of OFSP, free radical scavenging activity (µmol TE/kg fw) was reported to be 493–969 by Oki et al. (2006) samples from Japan, and in case of iron‐chelating ability, Rumbaoa et al. (2009) reported 310.7 (mg/mg, fw). In case of DPPH scavenging capacity, Shih et al. (2009) reported 889–1,372 (µg/ml) from OFSP in the Taiwan. Inhibition at 50 mg/ml of 92.0 was reported by Rumbaoa et al. (2009). Potato tubers with increased levels of flavonoids and high antioxidant capacities were developed by Lukaszewicz et al. (2004). According to Lachman, Hamouz, Orsák, Pivec, and Dvořák (2008) total polyphenolic acid and ACN content of different colored SP depend on the environmental factors and application of the fertilizer in agriculture practices.

## 11 ROLE OF OFSP IN HANDLING VITAMIN A MALNUTRITION

Black, Morris, and Bryce (2003) reported the role of retinol deficiency in night blindness, susceptibility to infections, cognitive development problems and immune system function, susceptibility to respiratory infection, diarrhea, measles, and malaria (Thurnham, McCabe, Northrop‐Clewes, & Nestel, 2003). UNICEF (2018) identified the retinol deficiency in 140 million children illness and death. WHO reported VDA is a critical community problem, and it influenced the health of one third of children aged from 6 to 59 months in 2013; children in sub‐Saharan Africa (SSA) (48%) and South Asia (44%) are the major affected. It also reported that 250,000–500,000 VAD children are in fate of blind in each year due to the vitamin A deficiencies, and 50% of them died within 12 months of vision loss (WHO, 2018).

One of the easiest ways to introduce more VA into the diet is by consuming the carotene‐rich plant‐based foods like OFSP. This is a good source of carotenoids, and they readily convert into retinol in human body. International Potato Center (2017) reported that supplementation of 100–150 g of the OFSP in human diet can prevent VAD in younger's and also radically diminish maternal mortality. Low et al. (2007) reported that in Mozambique 50 million children <6 year got benefited by consumption of WFSP along with OFSP. OFSP is popularizing to combat VAD in several SSA countries (Kapinga, Byaruhanga, Zschocke, & Tumwegamire, 2009), including South Africa (Faber, Venter, & Benadé, 2002; Laurie & Faber, 2008) through community‐dependent food intervention programs. In different strategies, incorporation of OFSP resulted in VA increase in different targeted groups (Haskell et al., 2004; Low et al., 2007).

Hortz et al. (2012) reported in their studies that introduction of OFSP in SSA countries such as Uganda and Mozambique is raised in VA intakes among children and women. Jones and de Brauw (2015) reported that biofortified OFSP in Mozambique facilitated the improved child health status and also reported the decrease in incidence and extent of diarrhea in children <5 years. Low et al. (2007) concluded that amplified production and utilization of the OFSP improved the nutritional status of the consumers in African countries, especially in Nigeria (Fetuga et al., 2013). With recent introduction of OFSP in East African countries such as Nigeria, peoples and government and nongovernment agencies concern on growth and utilization (Hagenimana & Low, 2000).

Still more efforts are required from the agriculture and health extension officers to encourage production and utilization of OFSP connecting households with young children in pastoral areas of VAD populations (Low et al., 2007). Prolonged storage of OFSP is usually achieved by drying (sun drying), and during the traditional drying (sun drying) techniques, microbial contamination was reported as the major problem due to the high moisture content of the fresh OFSP. Amajor et al. (2014) reported that OFSP flours can produce by sun drying and milling of the fresh OFSP roots. OFSP flour prepared by milling of dried slices can also utilize as entire flour or as a combined with others. Some of the reported studies on animal or human studies related to the role of OFSP in VAD management are summarized in Table 6.

**Table 6:** Some human and animal studied on the orange‐fleshed sweet potato (OFSP) in vitamin A‐related disease management

S. no.	Country/target group	Study	Results	Reference
1	Mozambique/Children	Longitudinal study reported from three deliberately selected districts, two are selected for intervention and one as control. Information was collected on demographic, agricultural, and anthropological issues. Blood samples were collected from all respondents for biochemical analysis	Intervention children reported higher intake of OFSP, reported high VA. Mean serum retinol level increased by 0.100 mM in intervention children, which are not increased in control subjects	Low et al. (2007)
2	Uganda/Children and women	Study was conducted to know impact of the intensive (IP) and reduced practices (RP) with a control on OFSP and VA intakes among children aged 6−35 months and 3–5 years and women, and IP compared with control on VA status of 3‐ to 5‐year‐old children and women with serum retinol <1.05 mM at baseline	9.5% point reduction in prevalence of serum retinol <1.05 mM identified. At follow‐up, VA intake from OFSP was correlated with VA status. Use of OFSP increased VA intakes of children and women	Hotz, Loechl, Lubowa, et al. (2012)
3	Bangladesh/women	Daily consumption of OFSP with or without added fat, on the VA status of Bangladeshi women with low initial VA status was done. Women received one of the following for 6 days/week over 10 weeks. 1. 10 mg RAE/day (WFSP and a corn oil) 2. 600 mg RAE/day (OFSP and a corn oil) 3. Fried OFSP and a corn oil capsule 4. Boiled WFSP and a retinyl palmitate capsule in addition to their home diets. Retinol and BC and VA were assessed before and after the 60 days	BC concentrations in plasma found in this study were high in groups consumed OFSP and plasma BC was higher in the consumed fried OFSP compared with boiled OFSP. Initial and final total body VA pool sizes were 0.060–0.047 mmol and 0.091–0.070 mmol. Concluded that, the impact of OFSP on VA status in Bangladeshi women was marginal	Jamil et al. (2012)
4	Mozambique/general consumers	Reported study on OFSP consumption. The two intervention models were compared: 1. Low intensity (1 year) 2. High‐intensity (nearly 3 years) training model	OFSP consumption raised VA among consumers. OFSP accounted for 47%–60% of all SP consummation provided 80% of total VA	Hotz, Loechl, de Brauw, et al. (2012)
5	South Africa/school children	Effect of boiled and mashed OFSP in improving VA levels in school children studied. Dewarmed 5–10 years kids were randomly assigned to following two sections for 53 days. 1. Treatment group consumed 125 g boiled and mashed OFSP 2. Control group given same portion of WFSP	High amount of VA reported in OFSP consumed group than control. The proportions of children with normal VA status in the treatment group increased and did not change in the control group	Van Jaarsveld et al. (2005)
6	Mozambique/children	Health benefits of biofortification in reducing VAD reported in rural area of north Mozambique	Children <5 years, biofortification reduced diarrhea prevalence by 11.4% and by 18.9% in children <3 years	Jones and de Brauw (2015)
7	Kenya/women	Role of OFSP nutrition and health‐promoting activity reported. VA intakes were assessed with multipass 24‐hr recalls in a subsample of 206 mothers at 8–10 months postpartum	22.9% of women had VA <1.17 mM. By 9 months of postpartum, intervention women had significantly higher intakes of VA‐rich OFSP in the previous 7 days	Girard et al. (2017)

## 12 PRODUCTS FORM OFSP

Orange‐fleshed sweet potato consumed frequently in family meal of different SSA countries by boiling, steaming, roasting, and drying (Low et al., 2007). Traditional sweet potato products are having significant role in income generation in small‐scale businesses and entrepreneurs run by women. In developing nations such as Uganda and other SSA countries, dehydrated and minimally processed foodstuffs from OFSP have recognized as the significant for domestic utilization and for small‐scale commerce in domestic markets (Sweet Potato Knowledge Portal, 2018). OFSP and their products are highly promoting in the different African countries such as Kenya, Uganda, Ethiopia, Mali by local governments and NGOs with the help of international research organizations (Abidin et al., 2015). Semiprocessed products from OFSP have been extensively studied in some SSA countries such as Kenya were reported. Complementary food in form of porridge by OFSP flour was highly accepted by the assessors (Stathers, Bechoff, Sindi, Low, & Ndyetabula, 2013). Researchers are concentrated on the methods to develop retention of the carotenes by the processing and are trying to develop the traditional foods by incorporating OFSP such as bread (Nzamwita, Duodu, & Minnaar, 2017), cookies (Kolawole, Akinwande, & Ade‐Omowaye, 2018), juices (Muhammad, Aminah, & Abbas, 2012), and porridge (Pillay, Khanyile, & Siwela, 2018) Table 7 shows the detailed explanation of different foods prepared by OFSP.

**Table 7:** Different food products from orange‐fleshed sweet potato (OFSP)

S. no.	Product group	Product	Description	Findings	Reference
1.	Baked products	Cookies	Cookies were produced from OFSP, mushroom powder with different blending ratios; only wheat flour was used as control	Cookies are identified with high protein, ash, crude fiber, and mineral as compared to control. Concluded that nutrient dense cookies with best sensory attributes can produce with blend OFSP and mushroom	Kolawole et al. (2018)
		Swahili Buns (*Mandazi*)	Developed and determine quality parameters in OFSP–wheat composite buns (*Mandazi*) at different levels of wheat flour substitutions with OFSP	Specific volume of the buns decreased significantly with increasing OFSP levels. Proximate composition, sensory acceptability, and VA content of product significantly increase by OFSP powder	Mongi, Simbano, Ruhembe, and Majaliwa (2015)
		Composite bread	Stability of BC during baking of OFSP–wheat breads	Baking causes the degradation of all‐*trans*‐BC, and breads containing 20% and 30% of OFSP flours can potentially be used for reducing the VAD in children	Nzamwita et al. (2017)
		Cakes	Evaluated the acceptance and preference of cakes prepared with OFSP flour	The cakes prepared with 40% OFSP flour had high acceptability among school students. Cake containing 40% OFSP can reach up to 22% of the RDA of VA to children between 10 and 13 years old	Rangel et al. (2011)
		Bread	OFSP–wheat flour enriched bread prepared and analyzed for different nutritional properties and BC	30% OFSP flour in bread can contribute 83.3 and 74.2% of VA to 3‐ to 6‐year‐old children's RDA	Kidane, Abegaz, Mulugeta, and Singh (2013)
		Sourdough *panettones*	OFSP flour on sourdough and prepared *panettones* by fermentation was reported. Also evaluated OFSP flour on the technological properties and volatile compounds of the final products	OFSP flour suitable for *panettones* with good moisture and strong yellow color with new volatile compounds such as 2‐octenal‐2‐butyl, dimethyl‐decane, and 2‐chlorooctane	Paula et al. (2018)
2.	Extruded products	Noodles	Noodles prepared up to 40% OFSP paste blending with domestic wheat flours and physical, chemical, and sensory properties were assessed	Noodles with OFSP showed quality of moisture and protein and concluded as OFSP is a promising for noodle ingredient	Ginting and Yulifianti (2015)
		Flours (extruded and nonextruded)	OFSP flour produced by extruded and nonextrusion. Effect of process on carotenoid contents of raw flours was determined	Extrusion reported stabilization of OFSP flours but reported in carotenoids losses due to moisture and screw speed with fixed screw configuration, barrel temperature, and formulation	Waramboi, Gidley, and Sopade (2013)
		Pasta	OFSP processed into flour using different processing methods with pretreatment (blanching, steaming, grilling) and produced pasta by extrusion	Pretreatments and processing methods had a significant effect on functional properties and the chemical properties of the OFSP. Concluded that OFSP could be used for pasta production with rich BC	Olubunmi, Abraham, Mojirade, Afolake, and Kehinde (2017)
		The extruded product with rice and OFSP flours	Evaluated and compared the total carotenoid content of two cultivars and the losses on the dehydrated extruded OFSP flour with different concentrations of rice flour	Losses of total carotenoids higher in the extruded products. The values of VA are very high, indicating that this product is a very good source of PVA. Total carotenoids content of 50% OFSP mixing with 50% of rice flour were reported very good source of PVA	Fonseca, Soares, Freire Junior, Almeida, and Ascheri (2008)
		Instant noodle	Evaluated the noodles prepared using wheat–OFSP–African yam bean flour	OFSP and African yam bean flour up to 20% and 30% resulted in a nutritious instant noodle	Effiong, Maduka, and Essien (2018)
3.	Dried products and flours	Powder (spray‐dried)	Determined effect of amylase and maltodextrin on OFSP pure drying. The nutrient composition and rheological properties of rehydrated powder determined	BC and vitamin C reduction reported. All‐*trans* form of BC was changed to *cis*. OFSP powders act like pregelatinized starch. Also concluded this product can use as thickener	Grabowski, Truong, and Daubert (2008)
		Flour (sun‐dried)	Microbiological, chemical, functional, and sensory properties of the fermented, sun‐dried OFSP flour determined	OFSP flours are microbiologically safe, and BC was reduced. Recommended a good processing method to retain PVA	Amajor et al. (2014)
		Dried chips (low temp.)	Determined the nature of PVA losses during drying at low temperature	16% and 34% reduction in *trans*‐BC. Both drying method and shape have effect on the BC. Sun drying slightly destroying PVA content compared to solar and hot air drying	Bechoff et al. (2009)
		Dried and stored OFSP	Preservation of carotenoids in OFSP chips determined. Impact of pretreatments to retain carotenoids after drying and storage for 6 months at room temperature was verified	Pretreated and soaked samples had higher content of carotenoid than the control. Also, researchers concluded that applying chemical pretreatment is effective	Bechoff, Westby, Menya, and Tomlins (2011)
4	Complementary and other foods	Complementary food	Assessed the acceptance of complementary foods made from OFSP	OFSP complementary food was well accepted in its color and soft texture, concluded that OFSP has the potential to use in complementary feeding to improve VA status	Pillay et al. (2018)
		Complementary food	Nutrient composition of OFSP complementary with maize–soybean–groundnut was done	OFSP complementary food is a good source of BC and VA status of infants. OFSP complementary food meets all the energy and macronutrient densities in the Codex	Amagloh and Coad (2014)
		Weaning foods	Effect of OFSP–cereal–legume blend of maize fortified with soybeans on weaning food determined	25% replacement levels with maize and OFSP are highly acceptable in nutritional	Bonsi, Plahar, and Zabawa (2014)
		Porridge (OFSP‐mangoes)	OFSP boiled and mixed with mangoes puree. Samples pasteurized (80°C for 5 min), packaged, hot filled, and cooled	BC and vitamin C loss identified after pasteurization. Sensory changes were reported after 6 months of the storage at room temperature	Muchoki and Imungi (2017)
		Blended foods	This study determined the BC degradation in the ready‐to‐eat OFSP‐derived products made under local processing conditions. The preparation (i.e., drying) and cooking process (either by boiling or frying) were conducted under noncontrolled conditions in Uganda	All‐*trans*‐BC contents were varied in porridges and chapati depends on processors. The all‐*trans*‐BC was greater in products cooked with oil, as compared to the boiled ones	Bechoff, Poulaert, et al. (2011)
		Complimentary food	OFSP‐based infant food developed in SSA with soybean, soybean oil, and fishmeal was processed as complementary food by oven toasting	The OFSP formulation meets energy, protein, fructose, and fat specifications but very less in amino acid compositions	Amagloh et al. (2012)
		OFSP and haricot bean food	Formulated foods from OFSP and haricot bean in different proportions and analyzed nutritional composition	Proximate composition, VA, and minerals are providing RDA for children. The food formulated from 70% OFSP and 30% haricot bean provides the highest protein, fat and fiber, energy, and minerals	Haile and Getahun (2018)
		Bambara groundnut–OFSP snacks	Ready‐to‐eat extruded snack prepared using a combination of OFSP and bambara groundnut	Snacks showed good source of nutrients for RDA for school children. Snack food is a good source of PVA with good sensory properties	Buzo, Mongi, and Mukisa (2016)
		Sweet potato *amala*	A traditional processing method of sweet potato flour for amala (a stiff paste meal) in Southwest Nigeria was done	Different methods resulted in sensory variations of *amala*	Fetuga et al. (2013)
5	Drinks	Radish and OFSP juice	Stability of color and pigment obtained from red radish and OFSP performed in a juice model system during 65 weeks	At room temperature, high stability was obtained in juices colored with C‐18 purified radish anthocyanins and lowest with OFSP. Refrigerated temperatures increased the half‐life of the pigment to more than one year	Rodríguez‐Saona, Glusti, and Wrolstad (1999)
		OFSP‐based juice drink	OFSP‐pineapple drink prepared from 50% to 10%, and different physico‐chemical and sensory properties were studies	OFSP‐based juice was prepared successfully with high overall acceptability. The drink also had substantial quantities of VA	Muhammad et al. (2012)
6	Other products	Natural colorants	OFSP anthocyanin‐based dye compared to synthetic red 40 and red 3 colorants as well as purple carrot and red grape commercial colorants	OFSP‐based dye has high‐to‐moderate resistance for pH, temp., and light. OFSP colors showed red‐violet hue for extended periods of time in comparison with synthetics	Cevallos‐Casals and Cisneros‐Zevallos (2004)
		Starch	The functional and structural properties of starches from six OFSP varieties were studied	Tainan 18 variety is good source of starch with high‐amylose with properties of high setback and breakdown viscosities, high water solubility at 85°C but low swelling volume at 65°C, and high hardness and adhesiveness	Lai et al. (2016)
		Bioethanol (raw materials)	Suitability to produce bioethanol of 50 varieties of the OFSP was determined	Reported that selected OFSP varieties are with good yields ranged between 23.6 and 49.0 t/ha and capable to produce ethanol between 3,320.1 and 5,364.5 L/ha	Waluyo, Roosda, Istifadah, Ruswandi, and Karuniawan (2015)

## 13 CONCLUSION

An eye pleasant color and good flavor make OFSP possesses acceptable by different age groups in many processed forms. The OFSP contained high moisture and starch contents; in contrast, protein and fats are present in very less concentrations. However, the ash and fiber contents are present in moderate concentrations. The minerals are moderately present in OFSP, calcium, magnesium, zinc, and sodium are reported in very less concentrations, but phosphorus and potassium are reported in moderate concentrations, and iron is reported in good concentrations. PVA and NPVA carotenoids are reported in the OFSP; in case of BCX, lutein, zeaxanthin, and AC and BC are in good concentrations. Among the all, very high concentrations of the BC are reported, which is a good source for the VA. Very low concentration of the Vit. E and Vit. C is reported in OFSP. In case of phenolic acids, such as CGA, CA, 4, 5‐; 3, 5‐; 3, 4‐diCQA isomers are reported in good concentrations. The presence of different carotenoids and phenolic acids with good AA is reported by different scientists. The role of OFSP is successfully reported in the VAM in developing countries. Scientists successfully prepared the different food products both in liquid and in solid forms by OFSP base. Finally, it is concluded that OFSP is a good secondary staple food for the developed countries.

## CONFLICT OF INTEREST

The authors declare no conflict of interest.

## ETHICAL STATEMENT

This study does not involve any neither human nor animal testing.
